# Anti-IAPP Monoclonal Antibody Improves Clinical Symptoms in a Mouse Model of Type 2 Diabetes

**DOI:** 10.3390/vaccines9111316

**Published:** 2021-11-12

**Authors:** Anne-Cathrine S. Vogt, Elisa S. Roesti, Mona O. Mohsen, Ainars Leonchiks, Monique Vogel, Martin F. Bachmann

**Affiliations:** 1 Department of Rheumatology and Immunology (RI), University Hospital, 3010 Bern, Switzerland; anne-cathrine.vogt@students.unibe.ch (A.-C.S.V.); Elisa.roesti@rmit.edu.au (E.S.R.); mona.mohsen@dbmr.unibe.ch (M.O.M.); Monique.vogel@dbmr.unibe.ch (M.V.); 2 Department for BioMedical Research (DBMR), University of Bern, 3008 Bern, Switzerland; 3 Latvian Biomedical Research and Study Centre, Ratsupites 1 k1, LV-1067 Riga, Latvia; ainleo@biomed.lu.lv; 4 Centre for Cellular and Molecular Physiology (CCMP), Nuffield Department of Medicine, The Jenner Institute, University of Oxford, Oxford OX3 7BN, UK

**Keywords:** islet amyloid polypeptide (IAPP), amyloid, type 2 diabetes (T2DM), monoclonal antibody (mAb), amylin

## Abstract

Type 2 Diabetes Mellitus (T2DM) is a chronic progressive disease, defined by insulin resistance and insufficient insulin secretion to maintain normoglycemia. Amyloidogenic aggregates are a hallmark of T2DM patients; they are cytotoxic for the insulin producing β-cells, and cause inflammasome-dependent secretion of IL-1β. To avoid the associated β-cell loss and inflammation in advanced stage T2DM, we developed a novel monoclonal therapy targeting the major component of aggregates, islet amyloid polypeptide (IAPP). The here described monoclonal antibody (mAb) m81, specific for oligomeric and fibrils, but not for soluble free IAPP, is able to prevent oligomer growth and aggregate formation in vitro, and blocks islet inflammation and disease progression in vivo. Collectively, our data show that blocking fibril formation and prevention of new amyloidogenic aggregates by monoclonal antibody therapy may be a potential therapy for T2DM.

## 1. Introduction

Type 2 diabetes mellitus (T2DM) is the most common form of diabetes, characterized by chronic inflammation of pancreatic islets, impaired insulin secretion, and the presence of amyloidogenic aggregates [1,2]. Numbers of T2DM patients are rising worldwide, reaching pandemic proportions; according to the International Diabetes Federation (IDF), 9% of adults aged 20–79 years—an astonishing 463 million people—are living with diabetes. In addition, if the current trends continue, a projection to the year 2045 estimates 700 million adults living with diabetes. Apart from the clear evidence that good lifestyle habits—comprising healthy diet [3] and moderate physical activity [4]—could decrease this high count, there is currently no available curative or preventive drug against T2DM. Approximately 50% of patients are diagnosed on time, and are able to slow the development of T2DM-associated organ damage with available conventional anti-diabetic drugs and ameliorated diabetes care. Nonetheless, symptomatic treatment alone does not stop the progression of T2DM to islet damage and other related irreversible complications [5].

In order to fill this void, new strategies need to be developed, targeting the pathophysiological mechanisms finally ending in β-cell failure and death [6,7]. Besides insulin resistance [8,9], which leads to more insulin demand and higher blood-glucose levels, we focused on the presence of amyloidogenic aggregates in the pancreatic islets which are mainly composed of accumulated islet amyloid polypeptide (IAPP), also known as amylin [1,10,11]. IAPP is a 37-amino acid polypeptide co-expressed and co-secreted with insulin from pancreatic β-cells [12]. Moreover, in vitro fibril, IAPP has the function of slowing gastric emptying, inducing satiety, decreasing food intake, and inhibiting glucagon, amongst other functions [1,13,14,15]. However, if over-produced, human IAPP is prone to aggregation, facilitated by its β-sheet monomeric structure that allows for the formation of hydrogen bonds when disposed in a parallel manner, resulting in β-stacks. If not eliminated on time, toxic and pro-inflammatory oligomer species are formed, eventually leading to β-cell failure [10,16,17,18]. Approximately 90% of T2DM patients have been shown to bear these amyloidogenic aggregates, and their presence is positively correlated with disease severity, as well as increased numbers of apoptotic cells and loss of β-cell mass [11,19]. Our strategy is to eliminate these pathogenic aggregates, and therefore delay or prevent T2DM. We have previously demonstrated that, by generating a virus-like particle conjugate vaccine against the N-terminal of human IAPP (VLP-N-terminal vaccine), the generated antibody (Ab) response had a high avidity for amyloidogenic aggregates, but a low affinity for soluble hIAPP [20]. This left the physiological response induced by IAPP intact, but prevented IAPP fibril formation, inflammation, and β-cell loss. To extend these findings to more controllable passive vaccination, we generated monoclonal antibodies (mAbs) that recognize oligomeric-, but not monomeric-, soluble hIAPP, and also recognizes pathogenic aggregates from T2DM human biopsies. Moreover, during in vitro fibril formation experiments, the mAb was able to prevent aggregation and block progression of aggregate formation when added during oligomerization. In addition, when injected in a transgenic mouse model mimicking T2DM in humans, the mAb was able to reduce hyperglycemia, fibril formation in the islets, and decreased inflammation, while insulin-production remained largely normal.

Thus, the here presented mAb is a good candidate for targeting toxic aggregates, and the delay or prevention of β-cell failure and the onset of T2DM.

## 2. Methods

### 2.1. Mice

FVB/N-Tg (Ins2-IAPP) RHFSoel/J mice [21] were purchased (Jackson Laboratory, Bar Harbor, ME, USA) at the age of 6 weeks, and bred in our facility, maintained as a homozygous colony on a standard chow diet (diet # 3430); (Granovit AG-Kliba Nafag, Kaiseraugst, Switzerland). All animals could acclimatize to the facility for one week before experiments were performed. After weaning, transgenic mice received a 45% kcal % high fat diet (diet #2126, Granovit AG-Kliba Nafag, Switzerland); Experiments were approved by The Animal Ethic Committee of the Swiss Cantonal Veterinary Office (Permission nr: BE70/18).

### 2.2. Pancreatic Human Tissue

Biopsies of patients’ pancreatic tissues with T2DM (under insulin treatment) were obtained from the University Clinic for Visceral Surgery and Medicine, Inselspital, Bern, Switzerland, with the approval of the Swiss Ethics Committees on Research Involving Humans (project ID number: 2019-00068).

### 2.3. Generation of Murine Monoclonal Antibodies by Hybridoma Technology

#### 2.3.1. Production of VLPs-Based Vaccine VLPs Obtained from the Coat Protein of the Bacteriophage

Qβ were expressed in *E. coli* strain JM109, with the expression vector pQβ10, and purified, as previously described [22]. The N-terminal IAPP peptide with disulfide bond (S-S) (H-KCNTATCATGGK[Aoa]-NH2 was purchased from Pepscan (Lelystad, The Netherlands), and chemically coupled to VLPs via the heterobifunctional S-4FB crosslinker (Solulink, San Diego, CA, USA), as previously described in Roesti et al. (2020) [20].

#### 2.3.2. Vaccination and Splenocytes Collection

Female C57BL/6 mice were immunized subcutaneously at 6 weeks of age with 10 μg of conjugated vaccine, followed by a boost injection on day 14. ELISA assays were performed as previously described [20] to check antibody titers. Splenocytes were further collected at and processed as described in the following section.

#### 2.3.3. Preparation of Myeloma Cell Culture, Feeder Cell and Cell Fusion

The following procedures were performed by ASLA Biotech. Sp2/0ag14 cells were fused by standard protocol with the IAPP-immunized mice splenocytes. Hybridoma selection was performed by using ELISA screening with RNase-coupled amylin peptide (5–10 μg/mL) as the antigen. After four rounds of selection, six clones with the highest absorbance (OD_492_ > 1) were selected for production and testing in ELISA. For production, hybridoma clones were kept in culture in the presence of DMEM glutaMAX^TM^ medium (Thermo Fisher Scientific, Waltham, MA, USA), supplemented with 10% Fetal Bovine Serum (FBS). The best clone obtained is named as m81 throughout the entire text.

#### 2.3.4. IgG Antibody Purification

The supernatant of cultivated hybridoma cells was collected and purified using a HiTrap Protein G HP antibody purification column (GE Healthcare Life Sciences, Chicago, IL, USA). As an equilibration and binding buffer, a self-made 20 mM sodium phosphate (pH 7.4) buffer was used, and for elution, a self-made 0.1 M glycine-HCl, (pH 2.8) was used. Fractions containing the eluate were pooled, neutralized with 1 M Tris HCl, and dialyzed against PBS (pH 7.4) for antibody storage. Absorbance at 280 nm was used to calculate protein concentration in the eluate fraction.

### 2.4. hIAPP Monomer and Fibrils Preparation

For monomer preparation, lyophilized peptide was freshly dissolved in hexafluoroisopropanol (HFIP), filtered in 0.22 μm-PVDF-Millex-GV filters (MerckMillipore, Burlington, MA, USA), and lyophilized overnight. Prior to each experiment, hIAPP was dissolved in ddH_2_O. To generate fibrils, the same hIAPP dissolved in ddH_2_O was kept at 37 °C overnight. To assess the presence of aggregates, a small amount was mixed with 50 μL of 60 μM Thioflavin T, and checked under the fluorescent microscope Axio Imager.A2, and a Carl Zeiss AxioCam.

### 2.5. Enzyme-Linked Immunosorbent Assay (ELISA)

Supernatants of cultivated hybridoma cells were tested for binding to IAPP. Corning 96 half-well plates were coated with 1 μg/mL RNase Peptide (S-S), 1 μg/mL hIAPP, and 1 ug/mL rIAPP in PBS overnight at 4 °C, undergoing shaking. Plates were washed with PBS, and blocked by addition of PBS-casein 0.15% for 2 h at room temperature to undergo shaking. Supernatants were 1:10 pre-diluted in PBS-casein 0.15% and further 1:3 were serially diluted. Plates were incubated 1 h at room temperature to undergo shaking, then washed with PBST (PBS + 0.05% Tween 20), and goat anti-mouse IgG-HRP (horseradish peroxidase) antibody (Jackson ImmunoResearch, Cambridgeshire, UK), diluted in PBS-casein 0.15%, was added to detect total IgG antibodies. Plates were incubated 1 h at room temperature while undergoing shaking, washed with PBST, and developed by addition of the tetramethylbenzidine (TMB) substrate. The developing reaction was stopped by 1 mol/L sulfuric acid, and plates were read at OD_450_ in a standard ELISA reader (BioTek Microplate Reader). The specificity of purified m81 mAb was tested using an identical setup and the same coating concentrations. Purified m81 was used at a start concentration of 100 ug/mL, and further at a 1:2 serial dilution in PBS-casein 0.15%.

### 2.6. Dot Blot

Nitrocellulose membranes (Merck Millipore, Schaffhausen, Switzerland) were plotted with 3 μL of 1 mg/mL of either rIAPP, hIAPP monomer, hIAPP fibrils, or a control amyloidogenic protein amyloid beta (Aβ; Bachem, Bubendorf, Switzerland). After drying, the membrane was boiled at 900 W for 5 min in a 4.3 mM Na_2_HPO_4_ 1.4 mM KH_2_PO_4_ containing a buffer (pH 7.2) to expose epitopes and increase antigen binding; it was then washed and blocked with 50 mM Tris-buffer containing 0.1% Tween20 and 2.5% casein. Overnight incubation with the purified m81 mAb was performed. Finally, a secondary anti-mouse IgG directly conjugated with Horse-Radish Peroxidase (Jackson ImmunoResearch, Cambridgeshire, UK) was used. Development of the membrane was performed using a SuperSignal^TM^ West Pico PLUS Chemiluminescent Substrate (Thermo Fisher Scientific, Basel, Switzerland), and pictures were acquired with an Azure C300 Imaging System (Axon Lab, Baden, AG, Switzerland). Fiji Image J was used to quantify the intensity of the dots. A background subtraction of 25 pixel was applied before quantification of the intensity or density. Monomeric IAPP and IAPP/Aβ fibrils were assessed with Thioflavin, as described above.

### 2.7. Immunofluorescence of Mouse and Human Tissue

Mice were sacrificed, and pancreas samples were collected and processed as previously described [20]. 1% Thioflavin S (T1892, Sigma-Aldrich, St. Louis, MO, USA) in ddH_2_O was used to stain for amyloid aggregates directly after deparaffinization. For the detection of β-cells, a rabbit mAb [EPR17359] IgG to Insulin (ab181547, Abcam, Cambridge, UK), followed by a goat anti-rabbit IgG conjugated to biotin (Nordic-MUbio, Susteren, The Netherlands) and a streptavidin conjugated Alexa546 (s11225, Molecular Probes, Eugene, OR, USA), were used. mAb IL-1β (F-5, sc-515598, Santa Cruz), followed by a Cy5-conjugated anti-mouse IgG (Jackson ImmunoResearch, Cambridgeshire, UK), was used to identified inflamed cells. For intracellular staining, sections were permeabilized with 0.5%-TritonX-100 in PBS. As a blocking solution bovine serum (3%), casein (0.5%) and NaN_3_ (0.1%) were prepared; however, when performing intracellular staining, the blocking buffer was made up of BSA (2%) and 0.5%-Triton X-100. In the presence of biotinylated Abs, an avidin/biotin blocking kit (Vector Laboratories, Burlingame, CA; USA) was used to block endogenous biotin according to the manufacturing instructions. All pictures were acquired with an Axio Imager.A2, and a Carl Zeiss AxioCam.

Quantifications were performed using Fiji Image J (NIH, USA), as previously described [20].

### 2.8. Thioflavin T Assay

Monomeric IAPP (prepared as described above) was dissolved in double distilled water (working concentration: 20 μM). Fresh and filtrated Thioflavin T (Sigma-Aldrich, St. Louis, MO, USA) was diluted in PBS (pH 7.4, working concentration was 60 μM). Purified m81 mAb was given at time = 0 and time = 100 min. Components were given in a black Nunc^TM^ MicroWell^TM^ 96-well Optica-Bottom plate (ThermoFisherScientific, Waltham, MA, USA), and the fibrillation process was measured at 37 °C in a Cytation5 Image Reader (BioTek, Winooski, VT, USA). The plate was shaken for 10 s prior to every read, at an excitation of 440 nm and an emission of 480 nm.

### 2.9. Monitoring and Immunization of the Transgenic Mice

After weaning, male homozygous hIAPP transgenic mice received a high fat diet (45% kcal from fat; diet # 2126; (Granovit AG-Kliba Nafag, Kaiseraugst, AG, Switzerland). From 6 weeks of age (day 0), mice received 100 μg of N-term(s-s) m81 mAb 2 times/week until the end of the experiment at alternating injection sites. Control groups received either PBS or 100 ug of IgG2a isotype control antibody (# BE0085, Bio X Cell). Weekly weight and 14-h fasting glucose levels were measured with an Accu-Check Aviva (Roche, Basel, Switzerland).

### 2.10. Glucose Tolerance Test (GTT)

GTT was performed according to Benedé-Uebito et al. [23]. Briefly, mice were pre-fasted for 6 h in the morning. Afterwards, basal levels of glucose were measured following intraperitoneal glucose injection (1.5 g glucose/kg body mass) of self-made 20% glucose in PBS (# 50-99-7; Sigma Aldrich, St.Louis, MO, USA). Blood glucose levels were measured 15, 30, 60, 90, and 120 min after glucose injection. The test was performed at weeks 6 and 14.

### 2.11. Insulin Tolerance Test (ITT)

ITT was performed according to Benedé-Uebito et al. [23]. Briefly, mice were pre-fasted for 6 h in the morning. Afterwards, basal levels of glucose were measured following intraperitoneal injection (0.75 IU/kg body mass) of self-made 0.25 IU Insulin in PBS (# 118-50; Cell Applications). Blood glucose levels were measured 15, 30, 60, 90, and 120 min after insulin injection. The test was performed for week 6 and 14.

### 2.12. Mouse IL-1β Enzyme-Linked Immunosorbent Assay

The ELISA was used for the quantification of interleukin 1 beta in the serum of HFD mice. The mouse IL1β/IL-1F2 (# DY401-05, R&D Systems^®^) DuoSet^®^ ELISA DEVELOPMENT SYSTEM kit was used as standard. 50 μL of serum was added to 96 well half- area ELISA plate (# 3690, Costar, Corning), which had been coated overnight at RT according to kit instructions. The ELISA was performed according to the kit instructions. For development, 50 μL of development solution (4.75 mL citrate buffer (30 mM), 0.25 mL 3,3′,5,5′Tetramethylbenzidine (TMB), and 25 μL H_2_O_2_ (30%)) was added to each well, and developed at room temperature for 20 min. After the incubation step, the reaction was stopped with 1 M H_2_SO_4_ (50 μL/well). OD_450_ was measured using the SpectraMax M5 ELISA reader (# M5, Molecular Devices) at a wavelength of 450 nm. OD_540_ was measured for wavelength correction.

### 2.13. Statistics

Statistical analyses were performed within two groups with the Mann–Whitney test, two-way ANOVA with post hoc Tukey’s HSD and Sidak’s multiple comparison when appropriated with GraphPad PRISM 9.0 (Graph-Pad Software, Inc., La Jolla, CA, USA). Statistical significance is displayed as *p* ≤ 0.05 (*), *p* ≤ 0.01 (**), *p* ≤ 0.001 (***), and *p* ≤ 0.0001 (****).

## 3. Results

### 3.1. m81 mAb Is Specific for Aggregated but Not Monomeric hIAPP

**Figure 1:**
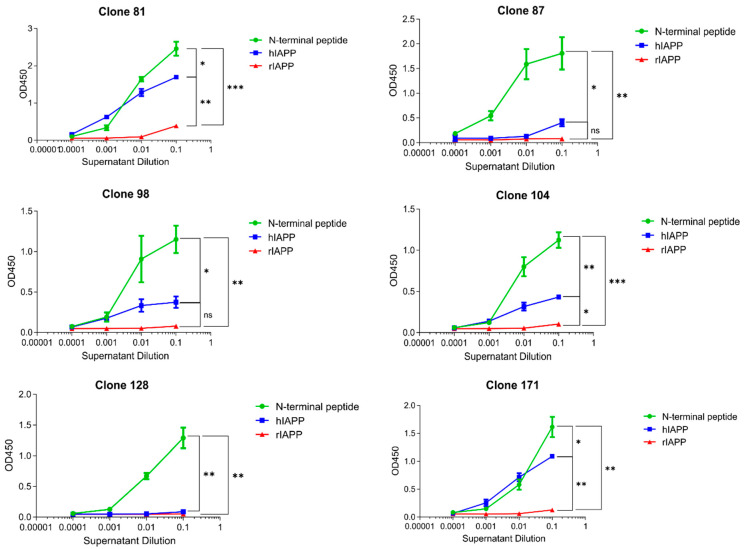
Binding specificity of different hybridoma clones. Different clones were tested in an ELISA assay with either RNase-Nterm(S-S) (green circle), hIAPP (blue square), or rIAPP (red triangle) coating. Data are presented as mean ± SEM. * *p* < 0.05, ** *p* < 0.01, *** *p* < 0.001.

**Figure 2:**
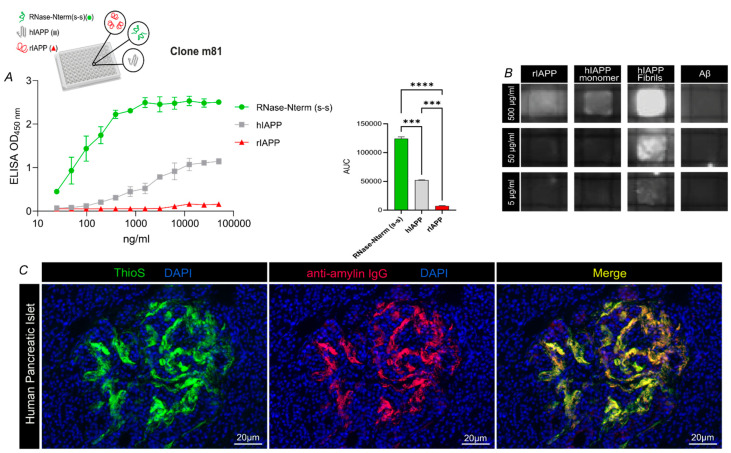
m81 mAb is specific for human IAPP fibrils, but not rat IAPP nor monomeric human IAPP. (**A**) Purified m81 mAb tested in an ELISA assay with either RNase-Nterm(S-S) (green circle), hIAPP (grey square), or rIAPP (red triangle) coating, with the corresponding area under the curve (AUC). Statistics were performed by one-way ANOVA using Tukey’s multiple comparison test, where the mean of each group was compared to the other groups. *** *p* < 0.001 and **** *p* < 0.0001. Data are presented as mean ± SEM. (**B**) Specificity for rIAPP, hIAPP monomer, hIAPP fibrils, and a negative control amyloidogenic Aβ peptide was tested on a dot blot at 500, 50, and 5 μg/mL. (**C**) Representative human pancreatic islet from a patient suffering from T2DM. Amyloidogenic aggregates were stained with ThioflavinS dye (ThioS, left panel, green) and purified m81 mAb(m81, middle panel, red). A merge image was created to visualize co-localization (Merge, right panel, yellow). RNase-Nterm(S-S), N terminal peptide with disulfide bridge (S-S); rIAPP, rat IAPP; hIAPP, human IAPP; Aβ, Amyloid beta; ThioS, ThioflavinS; T2DM. Scale bars: 20 μm.

We generated mouse hybridomas by immunizing mice against the N-terminus of hIAPP, as described earlier [20], according to standard protocols. Briefly, as shown in Figure 1, all hybridomas recognized the N-terminal peptide (RNase-Nterm(S-S)) of islet amyloid polypeptide (IAPP) [20] used for immunization, whereas, of six hybridomas tested, four were bound to aggregation-prone human IAPP (hIAPP), but none to rat (rIAPP), which failed to aggregate (Figure 1). hIAPP and rIAPP were both tested, as their final conformations are distinct due to the presence in the central peptide region of proline residues (Pro) in rat IAPP, but not human IAPP. Pro-residues acted as a β-sheet breaker, blocking aggregate formation. Hence, hIAPP forms aggregates, as it has no prolines, while rIAPP remains soluble. Interestingly, both human and rat IAPP share identical N-termini used for immunization, allowing for the direct comparison of human and rat IAPP binding by m81 mAb. One of the secreted hybridoma antibodies, m81, whose protein production was the highest, was chosen for affinity purification, and further characterized for antigen binding. In ELISA assays, purified m81 mAb recognized aggregated but not soluble, physiologically active IAPP, confirming the results obtained in the supernatants (Figure 2A). To further investigate the difference in recognition of IAPP monomers and fibrils by m81 mAb, a dot blot was performed by coating rIAPP, monomeric hIAPP, aggregated hIAPP, or a control amyloidogenic protein Aβ. m81 mAb clearly recognized aggregated hIAPP, but not rIAPP, soluble hIAPP, nor Aβ (Figure 2B), confirming the ELISA assay data.

We next examined whether m81 mAb could recognize the amyloidogenic aggregates present in severe T2DM patients. Pancreatic tissue was stained firstly with Thioflavin S (ThioS), to confirm the presence of pathogenic aggregates in Langerhans’s islets (Figure 2C, left panel). Second, we incubated sections with m81 mAb (Figure 2C), middle panel) to assess antibody’s specificity on human tissue. m81 mAb staining fully co-localized with ThioS staining (Figure 2C, right panel), confirming binding and specificity to hIAPP fibrils on pancreatic human tissue.

### 3.2. m81 mAb Prevents Fibril Formation and Stops the Aggregation Progress When Added at an Oligomeric State

**Figure 3:**
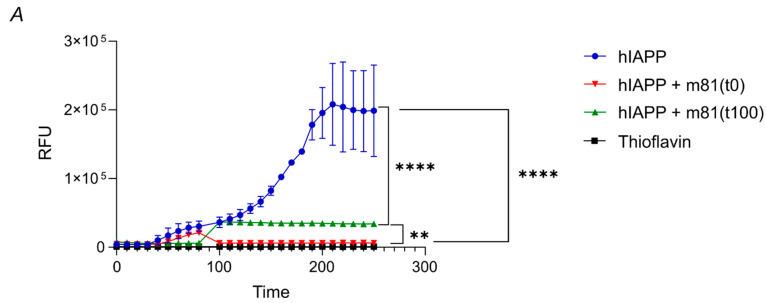
m81 mAb can prevent fibrils formation in vitro and block aggregation process up to an oligomeric stage. To investigate m81 mAb fibril blockage potential in vitro, synthetic human IAPP (hIAPP) was incubated at 37 °C, together with Thioflavin T, to detect the increase in fibril size. m81 mAb was either added at time point = 0 or 100 min. hIAPP, human IAPP; Thio, Thioflavin T. Data are presented as mean ± SEM. ** *p* < 0.01, **** *p* < 0.0001.

To investigate m81 mAb’s potential in preventing and stopping aggregate formation at different stages, m81 mAb was incubated in vitro with synthetic monomeric hIAPP or after in vitro hIAPP oligomer formation, together with Thioflavin T (ThioT), to quantify aggregation. When m81 mAb was added to soluble hIAPP before aggregation (Figure 3, hIAPP + M81(t = 0)), followed by incubation at 37 °C, hIAPP did not aggregate, in contrast to the control hIAPP (Figure 3, hIAPP). Interestingly, when m81 mAb was added after the synthetic fibrils had already reached an oligomeric state (at ca. 100 min, Figure 2, hIAPP + M81(t = 100)), the mAb stopped aggregate formation, and prevented further assembly into larger fibrils; under these conditions, Thioflavin T signal remained constant, indicating that previously formed aggregates cannot be reversed into the state of monomeric hIAPP. Thus, m81 mAb, by binding to oligomers, can prevent in vitro amyloid aggregate formation and block the progression of oligomers into larger fibrils, but cannot reverse oligomerization.

### 3.3. m81 mAb Is Able to Prevent Hyperglycemia, Fibril Formation in Pancreatic Islets, Decrease Inflammation and Maintain Insulin-Production in a Mouse Model of T2DM in Human

To investigate in vivo the ability of m81 mAb to recognize hIAPP aggregates and reduce aggregate load and damage in pancreatic islets, we took advantage of the existing transgenic mouse model FVB/Ins2-hIAPP, which expresses human IAPP under the rat insulin promotor. The phenotype of these mice closely resembles T2DM in humans, as male transgenic mice spontaneously develop diabetes mellitus by 8 weeks of age, which is associated with hIAPP deposits in the pancreatic islets, and associated with insulin resistance, leading to β-cell failure and death, as well as impaired insulin secretion and hyperglycemia

**Figure 4:**
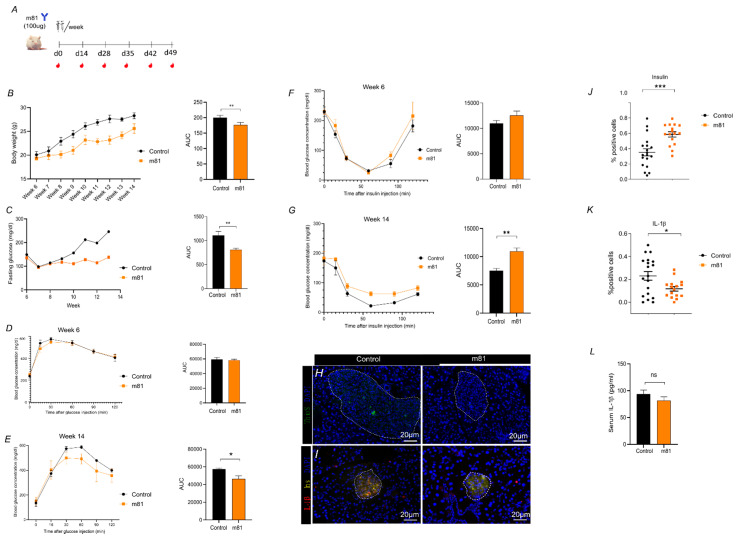
m81 mAb prevents hyperglycemia increase and human IAPP aggregate formation in pancreatic islets, and decreases IL-1β level. (**A**) Immunization scheme of FVB/Ins2-hIAPP transgenic mice with 100 μg of m81 mAb or control buffer for each injection. Mice received two injections/week. Blood was collected for Ab titer check. (**B**) Body weight [g] over 13 weeks of m81 mAb immunized (orange square) and control (black circle) mice, with corresponding area under the curve (AUC). (**C**) 14h-fasting glucose level [mg/dL] of m81 mAb immunized (orange square) and control (black circle) mice, with corresponding area under the curve (AUC). *n* = at least four mice per group. Statistics performed by unpaired t-test, where the immunized (m81 mAb) group is compared to the control (PBS) group. ** *p* < 0.01. (**D**) Glucose Tolerance Test at age of 6 weeks, with corresponding area under the curve (AUC). *n* = four mice per group. (**E**) Glucose Tolerance Test at 14 weeks of age, with corresponding area under the curve (AUC) *n* = four mice per group. Statistics performed by unpaired t-test, where the immunized (m81 mAb) group is compared to the control (Isotype) group. * *p* < 0.05. (**F**) Insulin Tolerance Test at age of 6 week, with corresponding area under the curve (AUC). *n* = four mice per group. (**G**) Insulin Tolerance Test at age of 14 week, with corresponding area under the curve (AUC). *n* = four mice per group. (**D**–**G**) Statistics performed by unpaired t-test, where the immunized (m81 mAb) group is compared to the control (Isotype) group. ** *p* < 0.01. (**H**) Pancreatic islets showing deposited hIAPP (ThioS, green) in control (left panel), m81 mAb immunized (right panel). Nuclei are stained in blue (DAPI). (**I**) Presence of pro inflammatory cytokine IL-1β (IL-1β, in red), insulin (Ins, in yellow), and nuclei (DAPI, blue) in control (left panel), m81 mAb immunized (right panel) (**J**) Quantification of insulin (left) and (**K**) IL-1β positive islets of control (black dot) and m81 mAb immunized (orange square) mice. *n* = 19 islets. (**J**–**L**) Statistics performed by unpaired t-test, where the immunized (m81 mAb) group is compared to the control (PBS) group. ** *p* < 0.01, *** *p* < 0.001. m81 mAb clone m81; hIAPP, human amylin; IL-1β, interleukin-1β; ThioS, ThioflavinS, Ins, insulin; DAPI, 4′,6-Diamidine-2′-phenylindole dihydrochloride. Scale bars: 20 μm. (L) IL-1β levels (pg/mL) in the serum of mice (*n* = 4) at 16 weeks of age determined by ELISA.

Male homozygous mice were injected twice per week with 100 µg of m81 mAb from week 6 until week 14 (Figure 4A), while fasting glucose levels and body weight were monitored. Starting from week 9, the control group showed an increased fasting glucose level, together with an increased body weight (Figure 4B,C), while the m81 mAb-treated mice maintained normoglycemic levels. A total of eight weeks (week 14) after treatment, this stable fasting glucose level in mAb treated mice was accompanied by a reduced glucose area under the curve during the glucose tolerance test (Figure 4E). In contrast to the glucose tolerance test, the insulin tolerance test (Figure 4F,G) resulted in accelerated clearance of glucose in control mice at week 14 (Figure 4G). This somewhat unexpected finding might be explained by the fact that the control mice move on from Type 2 to Type 1 diabetes, as they showed reduced rather than enhanced insulin production (Figure 4J). Hence, the control mice, in contrast to the m81 mAb treated mice, which show still near normal insulin production, respond to insulin in an enhanced fashion and glucose levels become more profoundly reduced upon insulin injection. These data pinpoint an increased insulin sensitivity, allowing mAb treated mice to absorb more glucose than mice treated with the isotype antibody control. At week 14, mice were euthanized, and their pancreas was collected for the presence of hIAPP aggregates, total insulin-content of β-cells, and inflammation levels, which were quantified histologically. Pancreatic slides of mice treated with m81 mAb showed significantly decreased levels of amyloidogenic aggregates detected with Thioflavin S (Figure 4H–J), along with higher insulin (Figure 4H–J) and lower IL-1β levels (Figure 4H,I,K). We also observed a trend of decreased IL-1β in serum, though not statistically significant, in mice treated with m81 mAb (Figure 4L). This indicates that treatment with m81 mAb can reduce endogenous hIAPP aggregates in vivo, and ameliorate symptoms of T2DM.

## 4. Discussion

Amyloid aggregates can be found extracellularly in pancreatic islets, and are principally made of islet amyloid polypeptide (IAPP). Aggregated IAPP have cytotoxic properties [24,25] and their presence is associated with β-cell failure, loss of β-cells, and correlates with severity of T2DM in affected patients [15,26,27]. Recently, it has been demonstrated that impaired intracellular turnover of IAPP by proteasomes and autophagy contribute to the accumulation of toxic IAPP, leading to β-cell death during T2DM [28,29]. Therefore, effective therapeutic strategies for disease modifying treatment of T2DM are urgently needed. Hence, targeting aggregated IAPP may therefore be an attractive axis to follow for a potential mAb therapy. Thus, we aimed at generating a mAb that specifically recognizes aggregated, but not soluble, IAPP. We have recently described a vaccine that induces this type of antibodies [20], and may be used to therapeutically immunize to treat T2DM. We therefore used the same vaccine to immunize mice and generate mAbs. One hybridoma antibody, m81, was selected, which indeed had the desired properties of recognizing oligomers and amyloid fibrils, but not soluble IAPP. Importantly, m81 also recognized aggregated IAPP in the pancreas of patients with T2DM. Furthermore, this mAb was able to block IAPP aggregate formation in vitro, and reduce aggregate loads in vivo in a murine model of T2DM. Furthermore, treatment with m81 mAb also reduced symptoms of T2DM, such has pancreatic inflammation, hyperglycemia, and glucose intolerance. Thus, m81 mAb may represent a first in class disease modifying drug against type T2DM.

The here-employed transgenic mouse model of T2DM develops disease very rapidly, within a few weeks. Correspondingly, it is difficult to start treatment long after onset of disease. This is very different for the disease in humans, where T2DM develops over years or decades. Hence, truly therapeutic treatment in humans with a humanized m81 is more realistic than in this particular mouse model.

We demonstrate here that m81 mAb is able to block IAPP oligomerization and fibril formation in vitro. This is likely also an important mechanism of action for m81 mAb in vivo: prevention of aggregate formation. As aggregates have a natural turn-over, prevention of new aggregates in the presence of natural aggregate removal may change the equilibrium towards reduced presence of aggregates. It has been shown for Aβ-deposits in Alzheimer’s plaques that Abs may promote plaque removal via Fc-receptor mediated uptake by myeloid cells. This is likely also the case here, and IAPP-aggregate specific Abs may promote aggregate uptake by inflammatory macrophages via Fc-receptors [30]. As Fc-receptor engagement often causes cellular activation, it will be interesting to see whether m81 mAb that fails to engage Fc receptors may promote aggregate removal under condition of reduced inflammation.

This therapeutic strategy of targeting IAPP by treatment with m81 mAb may not only be interesting for T2DM, but also for T1DM, in particular for the case of pancreatic islet transplantation. Specifically, there is a risk of β-cell failure after transplantation over time, largely due to amyloidogenic IAPP-aggregate formation [31]. Thus, blocking IAPP aggregation by m81 mAb after islet transplantation may reduce the risk of subsequent β-cell failure.

## 5. Conclusions

Here, we describe a novel IAPP-specific mAb which specifically recognizes aggregated, but not soluble, IAPP. This antibody is shown to prevent IAPP aggregation in vitro and aggregate deposition in vivo, delaying the onset of T2DM. Thus, subsequent to humanization, the here-described mAb m81 may offer a first in class therapeutic modality in T2DM patients.

## Data Availability

The datasets generated during and/or analyzed during the current study are available from the corresponding author on reasonable request.
